# Molecular insights and cell cycle assessment upon exposure to Chaga (*Inonotus obliquus*) mushroom polysaccharides in zebrafish (*Danio rerio*)

**DOI:** 10.1038/s41598-020-64157-3

**Published:** 2020-05-04

**Authors:** Jehane Ibrahim Eid, Biswadeep Das

**Affiliations:** 1 Department of Zoology, Faculty of Science, Cairo University, Giza, 12613 Egypt; 2 School of Biotechnology, KIIT University, Bhubaneswar, 751024 India

**Keywords:** Environmental biotechnology, Molecular engineering, Cell division, DNA damage and repair, DNA replication

## Abstract

Chaga (*Inonotus obliquus)* mushroom is considered as one of the most powerful antioxidants across the world. Though the therapeutic effects of Chaga components are well characterized *in vitro*, the *in vivo* developmental effects are not elucidated in detail. In this study, we assessed the *in vivo* developmental effects of Chaga polysaccharides in zebrafish, along with revealing the effects on cell cycle and apoptosis. Chaga mushroom polysaccharides comprised xylulose, rhamnose, mannose, glucose, inositol, and galactose, in addition to phenolic compounds; zebrafish embryos exhibited normal embryonic development upon transient exposure to Chaga extract (24 hours). Most embryos (>90%) were found to be healthy even at high concentrations (5 mg/mL). In addition, staining with the DNA binding dye, acridine orange showed that Chaga polysaccharides alleviated oxidative stress. Flow cytometric analysis using H_2_DCFDA that specifically binds to cells with fragmented DNA showed significantly reduced levels of intracellular reactive oxygen species (ROS) (p < 0.05), which in turn reduced apoptosis in the developing embryos. Cell cycle analysis by measuring the DNA content using flow cytometry revealed that Chaga polysaccharides moderately arrested the cells at G1 stage, thereby inhibiting cell proliferation that can be further explored in cancer studies. Overall, transient exposure of Chaga polysaccharide extract reduced intracellular ROS and assisted in the normal development of zebrafish.

## Introduction

Modern medicine is undergoing a paradigm shift towards personalized medicine and the adoption of natural therapeutics, such as biocompounds. The National Cancer Institute (NCI), United States has recently intensified its focus on natural products such as plants, marine organisms, and certain microorganisms for use in drug and biosimilar discovery^1^. One such medically important agent is *Inonotus obliquus* or Chaga mushroom that comprises compounds possessing anti-inflammatory, anti-tumor, and analgesic effects, in particular the low molecular weight polysaccharides. Chaga mushroom belongs to the family Hymenochaetaceae of Basidiomycetes, and is a rare edible fungus that mainly thrives in extreme cold temperatures down to −40 °C^2^. Chemical analysis of Chaga mushroom demonstrated that it comprises polysaccharides, triterpenes, polyphenols, melanin, and steroids, exhibiting several beneficial biological activities such as immunomodulatory and anti-cancer activities^3^. Chaga extracts have been used in Korea, China, Russia, Japan, and Siberia for their beneficial effects on lipid metabolism and cardiac function, as well as for antibacterial, anti-inflammatory, antioxidant, and antitumor activities^4,5^. Furthermore, aqueous extracts of Chaga, particularly polysaccharides exhibited anti-inflammatory effects in animal models of colitis and enhanced lipid metabolism^6^. Chaga-derived polysaccharides exhibited protection against diabetes and hepatic diseases^7,8^. Chaga is popular in many countries as its extract is widely used as tea for several remedies, in particular, for gastric problems and also in folk medicine for treating tumors^4^. Therefore, Chaga-derived polysaccharides are considered to be one of the valuable sources of antioxidant and antitumor compounds^9^. However, detailed investigations are needed to explore the underlying mechanisms for their therapeutic use. Although several studies have characterized the growth and metabolic dynamics *in vitro*^10–14^, its effects on *in vivo* model systems, particular on the growth and developmental dynamics is yet to be clarified prior to going through clinical trials. Furthermore, most of the studies assessed the antitumor role of Chaga mushroom using *in vitro* and early stage models through acute exposure experiments^4,10^, and so, warrants more long-term exposure experiments in higher animal models for delineating its potential effects under clinical conditions.

In this regard, zebrafish is a fascinating biological model for studying the embryonic development upon exposure to several environmental agents owing to more than 70% genomic DNA orthology with humans, small size, rapid development cycle, transparent embryos, ability to exhibit phenotype plasticity, robustness, amenable DNA repair system, and inexpensive to maintain compared to mice. Zebrafish has also been used as a eukaryotic model organism in toxicological and developmental biology studies because zebrafish exhibit several toxicological end points at cellular levels that can be measured and quantified^15,16^. Hence, elucidating the role of Chaga mushroom polysaccharides on zebrafish development and its effects on the cell cycle will reveal the underlying mechanisms of Chaga mushroom polysaccharides in modulating the growth dynamics *in vivo*. In this study, we have performed *in vivo* molecular characterization of Chaga mushroom polysaccharide extract on the developmental dynamics and cell cycle analysis in the early stages of zebrafish for assessing the effects of Chaga mushroom on early development.

## Results

### Chaga mushroom polysaccharide component analysis using GC-MSMS

**Figure 1:**
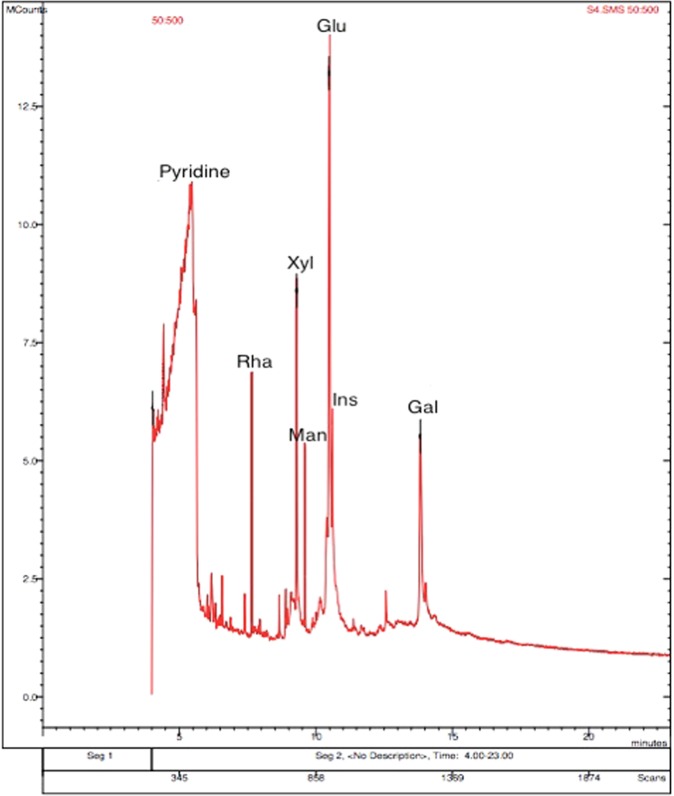
GC-MS chromatogram depicting the retention time peaks for different monomers of Chaga mushroom polysaccharides after extraction using hot water method.

Chaga mushroom crude polysaccharide was hydrolyzed and analyzed through GC-MSMS using silylating agent, and revealed that the different components of crude polysaccharide extract were obtained at different retention times, corresponding to unique monomeric compounds. The main monomeric components (retention time) were xylulose (7.7), rhamnose (9.3), mannose (9.9), glucose (12.3), inositol (12.5), and galactose (14.3) (Fig. 1). In addition to these components, many other minor components like phenolic compounds were also found to be present in trace amounts. The presence of such diverse monomeric components that are the constituents of the major fungal polysaccharides like β-glucans could be attributed to the versatility of the biological implications of Chaga mushroom.

### Zebrafish developmental analysis upon Chaga polysaccharide extract exposure

**Figure 2:**
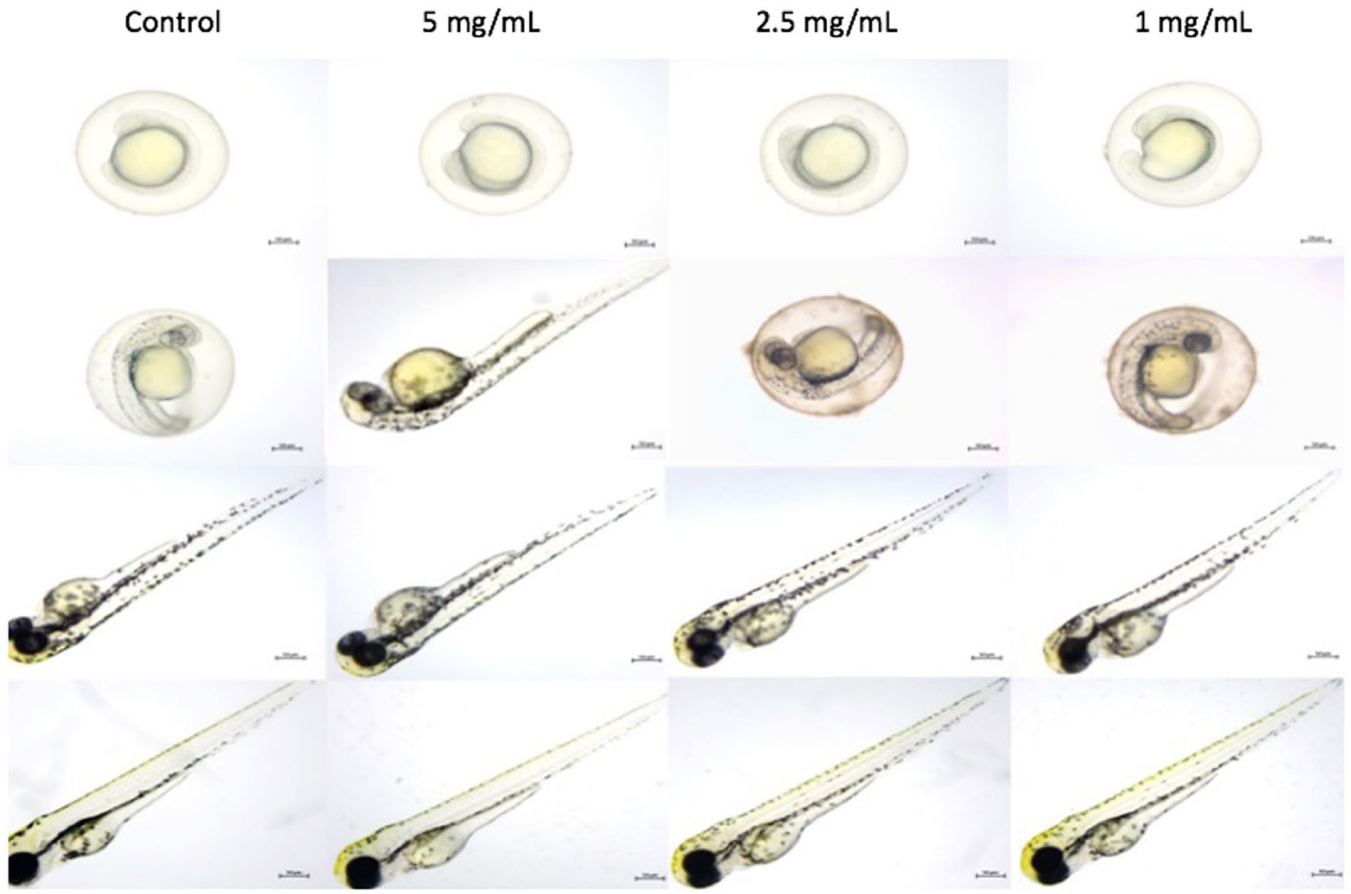
Bright field images of morphological analysis of zebrafish embryos (1 dpf to 4 dpf) showing normal development in control and Chaga-exposed zebrafish embryos.

The vital parameters, such as survivability rate, hatching rate, and morphological features did not vary much among the zebrafish exposed to different concentrations of Chaga mushroom polysaccharides in comparison to control. Early hatching was recorded in a few zebrafish exposed to high concentration of Chaga mushroom polysaccharides (5 mg/mL), though the difference was not significant across the groups. We did not observe any morphological deformities such as spinal curvature, yolk sac edema or tail malformations in any of the groups. There was not much difference in the heartbeat rate in the test groups in comparison to control. Overall structural manifestations were similar to that in the control fish (Fig. 2) that depicted Chaga mushroom polysaccharides did not induce developmental defomities during the whole experimental period (up to 7 dpf).

### Cell cycle assessment

Cell cycle analysis by measuring the DNA content of single cell suspension using the forward side scatter (FSC) height versus area parameter of the flow cytometer using the DNA binding-PI staining dye showed that the cells of the zebrafish exposed to Chaga mushroom polysaccharides and control depicted relatively similar peaks for the G0/G1 (corresponding to peak 3), S (corresponding to peak 4), and G2 (corresponding to peak 5) phases; however, the percentage of cells in the G2 was moderately less (16.1%) in comparison to control (24%) (Supplementary Figs. 1 and 2). This showed that Chaga mushroom polysaccharides arrested the cells mostly at G1, and hence checked cell proliferation. This anti-proliferative property of Chaga mushroom polysaccharides could be further utilized *in vivo* and *in vitro* cancer studies.

### Qualitative apoptotic analysis using fluorescence microscopy

**Figure 3:**
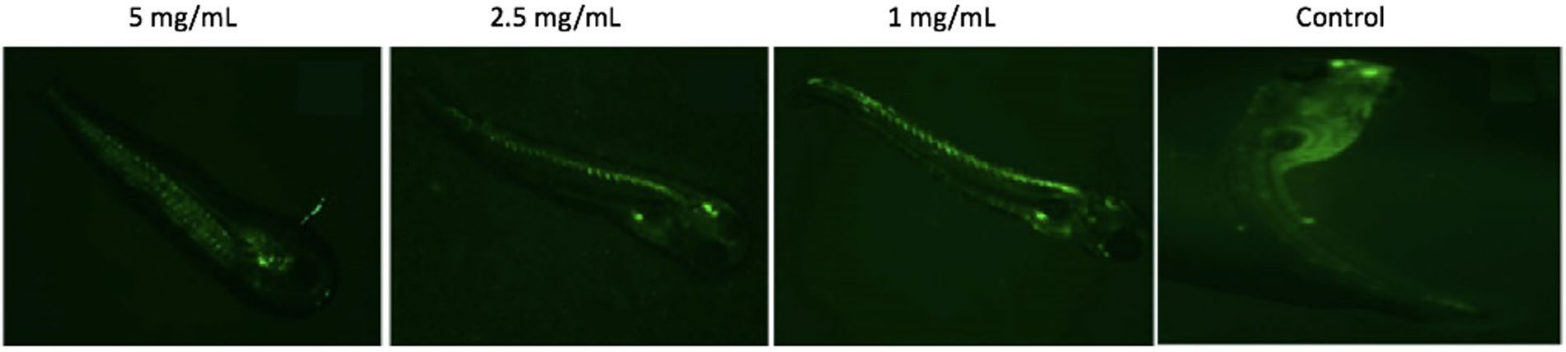
Apoptosis analysis of zebrafish embryos (5 dpf) grown in different concentrations of Chaga mushroom polysaccharides and determined by acridine orange (AO) staining, showing intensive green fluorescence as significant uptake of AO dye due to cellular-compartment damage in the cells.

Apoptotic assay using acridine orange staining revealed that apoptosis (as green dots) was induced less in the zebrafish exposed to higher concentrations of Chaga mushroom polysaccharides (2.5 and 5 mg/mL) compared to control (Fig. 3). This result is interesting and could suggest that Chaga polysaccharide components have anti-oxidative properties, by possibly neutralizing free radicals generated, thereby inhibiting apoptosis and protecting DNA from oxidative damage.

### Quantitative apoptotic analysis using Annexin-V/FITC and PI staining

**Figure 4:**
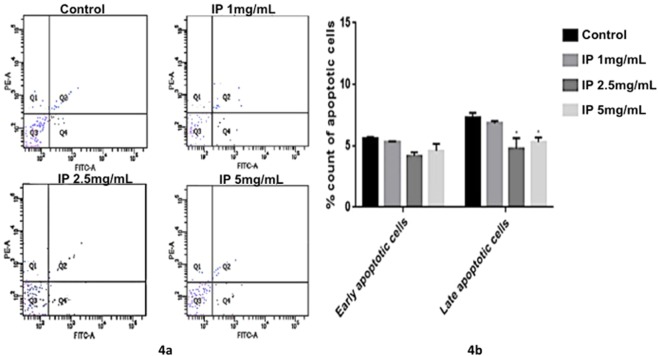
Annexin/PI apoptosis analysis in zebrafish embryos (5 dpf) grown in different concentrations of Chaga mushroom polysaccharides (control, 1 mg/mL, 2.5 mg/mL and 5 mg/mL). 4a. The proportion of non-apoptotic cells (Q3: Annexin V-FITC−/PI−), early apoptotic cells (Q4: Annexin V-FITC + /PI−), late apoptotic/necrotic cells (Q2: Annexin V-FITC + /PI + ) and dead cells (Q1: Annexin V-FITC−/PI + ) are shown. 4b. Compared to control, the percentage early apoptotic cells (Q4 quadrant of the flow cytometric graph) were found to be moderately lesser in 2.5 mg/mL and 5 mg/mL Chaga polysaccharide treated zebrafish embryos. For late apoptotic cells (Q2 qaundrant of the flow cytometric graph) significant differences (p < 0.05) were observed among control and test groups (2.5 mg/mL and 5 mg/mL). Values represent mean ± SD for three independent experiments. *p < 0.05 denote significant variation from control embryos as obtained by ANOVA analysis.

For the quantification of apoptosis, we performed dual Annexin-V/FITC and PI staining of control and zebrafish exposed to different concentrations of Chaga mushroom polysaccharides at 72 hpf (Fig. 4). The externalization of phosphatidylserine of the plasmalemma can be detected by Annexin-V/FITC in flow cytometry, whereas the integrity of plasma membrane is checked by propidium iodide (PI). Annexin-V and PI cannot stain the viable cells, whereas Annexin-V positively stains the early apoptotic cells but not PI. As the cascade mechanism of apoptosis progress, the late apoptotic cells are positively stained by both Annexin-V and PI. In case of necrosis, cells are positively stained by PI, but not Annexin-V. Flow cytometry results showed that control embryos and Chaga extract-treated embryos exhibited similar apoptotic profile; Chaga-extract treated embryos further showed lower number of apoptotic cells in a concentration dependent manner (Table 1; Fig. 4). All these results further supported acridine orange staining data observed in the study, depicting that Chaga mushroom polysaccharides exhibited anti-apoptotic activity.

**Table 1:** Table showing the percentage of viable cells, early apoptotic cells and late apoptotic cells of zebrafish embryos (5 dpf) upon exposure to different concentrations of Chaga polysaccharide extract after Annexin-PI flow cytometric analysis.

Exposure groups	Viable cells (% ± SD)	Early apoptotic cells (% ± SD)	Late apoptotic cells (% ± SD)
Control	80.1 ± 1.4	6.41 ± 0.7	8.1 ± 0.7
1 mg/mL	76 ± 1.7	5.21 ± 0.23	6.43 ± 0.04
2.5 mg/mL	83.2 ± 1.23	3.9 ± 0.03	3.7 ± 0.04
5 mg/mL	84.2 ± 1.6	4.5 ± 0.07	4.8 ± 0.04

### Intracellular ROS analysis

**Figure 5:**
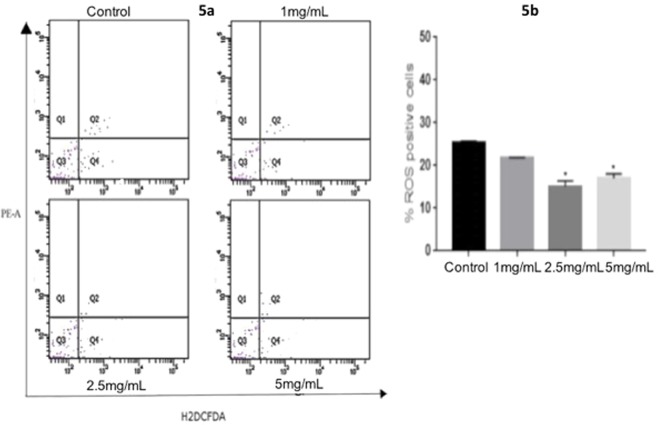
ROS analysis in zebrafish embryos (5 dpf) using flow cytometry: 5a. Q4 represents the ROS + level measured by H_2_DCFDA fluorescence dye. 5b. Bar graph showing the percentage of ROS + cells (Q4 quadrant of the flow cytometric graph) in zebrafish embryos grown under different concentrations of Chaga mushroom polysaccharides. Mean value ± SD of ROS positive cells were found to be significantly reduced (p < 0.05) in the test groups (2.5 mg/mL and 5 mg/mL) compared to control. Values represent mean ± SD for three independent experiments. *p < 0.05 denote significant variation from control embryos as obtained by ANOVA analysis.

Intracellular ROS analysis using H_2_DCFDA showed no/minimum variations in the mean DCFDA fluorescence in the zebrafish exposed to different concentrations of Chaga mushroom polysaccharides and control. This finding showed that ROS generation was inhibited by Chaga mushroom polysaccharides. Representative FACS plots showing the dissociated zebrafish embryonic cells subjected to different concentrations of Chaga mushroom polysaccharides are shown in Fig. 5.

### Glutathione S-transferase (GST) analysis

Analysis of glutathione S-transferase (GST) enzyme levels in the homogenized cells of zebrafish exposed to different concentrations of Chaga mushroom polysaccharides did not show significant differences in the mean GST activity compared to control. This finding suggested that Chaga polysaccharides did not induce metabolic stress in the zebrafish embryos and maintained the antioxidant defenses of the cells without any disturbances.

## Discussion

The recent surge in medicinal mushrooms because of their unique inherent beneficial attributes such as anti-inflammatory, anti-tumor, anti-oxidant, and anti-diabetic properties has been noteworthy^10,14,17,18^. Chaga mushroom is one such mushroom that is considered to be one of the highest anti-oxidant compounds^19^ and possess several such beneficial properties. In the present study, we showed that the hot water extract of the Chaga mushroom polysaccharides significantly reduced apoptosis and the generation of intracellular ROS that protected the cellular DNA from oxidative damage, in addition to enhancing the development of zebrafish. The major polysaccharide ingredient of Chaga mushroom is β-glucan, a polymer of β-D glucose and has been reported to possess several health benefits^20,21^. β-glucan mostly acts like a dietary fibre that is not digested in the human upper gastrointestinal tract, and greatly aid the body in digestion and absorption. It has been reported to have anti-diabetic, cholesterol reducing, and anti-proliferative properties^22–25^. The present study revealed the presence of unique monomeric units such as xylulose, rhamnose, inositol and glucose, some of which are the components of β-D glucans, as well as possess similar properties to that of β-D glucans. Therefore, such diverse beneficial properties of Chaga mushroom could be attributed to its sugar components. Besides, several other substances such as phenolic compounds present in Chaga extract could also contribute to the beneficial attributes of Chaga mushroom that need to be explored further.

In this study, DNA content analysis using flow cytometry demonstrated that zebrafish cells were moderately arrested at G0/G1 phase of the cell cycle upon exposure to Chaga polysaccharide extract, which is in line with several reports that show Chaga extracts induce G0/G1 cell cycle arrest^26,27^. Although the effect was less pronounced in the healthy zebrafish cells in the present study, this attribute can have tremendous implication in cancer studies, and can thus be a potent strategy to look into for checking the proliferation of cancer cells. Several studies have also reported the extended G1 phase period in zebrafish cells after midblastula transition (around six hours post fertilization) upon exposure to certain agents owing to the expression of zygotic genes and rigorous cell-cycle regulation^28,29^ that also supports our findings. Furthermore, the same cell-cycle inhibitory effects are much more pronounced in cancer cells upon treatment with Chaga mushroom polysaccharides^27^; the underlying mechanisms need to be still further clarified. The eukaryotic cell cycle is regulated by signal transduction pathways mediated by a sequential series of cell-cycle regulators, called Cdks, whose activation is dependent upon their association with cyclins^30^. The arrest of the cells at G1-phase provides an opportunity for cells to either undergo repair mechanisms or proceed through the apoptotic pathway^31^. Studies have documented that Cdk activities regulate G1/S transition in mammalian cells^32^. In addition, researchers have reported that Chaga mushroom extract have caused the reduction of cyclin or Cdk expression that resulted in the blocking of cyclin/Cdk complex formation and that lowered the levels of another protein pRb, which is required for the cells to enter to S phase^26,33^. Hence, our observation of increased number of cells in the G1 phase of the Chaga ploysaccharide-treated cells could be explained by the possible reduction of Cdks and cyclins that eventually arrested the cells at G1 phase. Interestingly, Chaga mushroom extracts have long been used as tea for treating tumors and gastric issues in folk medicine, and thus, our findings could possibly unravel the mechanism underlying such beneficial effects^14,18^, which can further be implicated in clinical trials.

Biocompatibility of a compound *in vivo* needs to be elucidated prior to clinical trials. Biocompatibility of a compound depends on its cytotoxic effect that can be inferred by assessing its morphological, cellular, metabolic, and molecular changes due to their exposure to live models^15^. Developmental deformities and oxidative stress are such phenomena that are widely assessed to study growth and metabolic dynamics^34^. *In vivo* developmental analyses in the present study showed that Chaga mushroom polysaccharide extract did not show any significant developmental changes in the exposed zebrafish embryos compared to control. Interestingly, several embryos demonstrated faster development when exposed to Chaga mushroom polysaccharides at high concentration (5 mg/mL). This finding showed that Chaga mushroom assisted in enhancing the embryonic development. Biocompatibility assays of Chaga mushroom polysaccharides on zebrafish embryos demonstrated that Chaga mushroom had a negative impact on oxidative stress and reduced the progression of apoptosis, even at higher doses (5 mg/mL) (Fig. 5). DNA binding dye AO staining dye demonstrated less number of apoptotic cells in the polysaccharide-treated group, thereby demonstrating the anti-apoptotic nature of Chaga mushroom polysaccharides, which can thus lower oxidative stress. Cellular biocompatibility of the polysaccharide extract was further confirmed by assessing the intracellular ROS and antioxidant enzyme status. Chaga mushroom was found to reduce the number of intracellular ROS, and did not affect the levels of antioxidant enzymes. This finding shows that Chaga mushroom polysaccharide components could quench the free radicals/oxidative species generated in the cell and can thus act as an anti-oxidant molecule. Furthermore, it reduced the overall burden of oxidative species, thereby maintaining the level of antioxidant enzymes, rendering the cells to be free of metabolic stress. The present study is the first to comprehensively demonstrate the effect of Chaga mushroom polysaccharides on the growth and development of zebrafish. The findings suggested that Chaga mushroom polysaccharides enhanced the embryonic development and reduced oxidative stress in the developing embryos, and therefore could be a useful potential natural compound to mitigate oxidative stress-related disorders.

## Methodology

### Chaga mushroom polysaccharide extraction and purification

#### Hot water-based extraction of polysaccharides from Chaga mushroom

Raw fresh Chaga mushroom was purchased commercially (Siberian), ground to fine powder and dissolved in water (10 g in 150 mL water) to prepare a homogenous solution, and refluxed twice for 2 hours at 70 °C with water using reflux condenser and hot water bath. The solution was then filtered using Whatman No. 3 paper and the filtrate (approx. 100 mL) was concentrated using a rotavapor at 40 °C, and dissolved in 3 volumes of 95% ethanol, followed by reflux for 2 hours at 50 °C. The concentrated extract was then centrifuged at 5000 rpm for 10 min, and the supernatant was obtained and dried using rotavapor at 50 °C for obtaining crude polysaccharide extract. To remove proteins from the crude polysaccharide, Sevag reagent (chloroform:butanol in the ratio 4:1) was added to the crude polysaccharide. Briefly, the crude polysaccharide was dissolved in sterile water (10 mg/mL). Sevag reagent was added to the sample in the ratio of 1:1, and subsequently mixed and vortexed thoroughly. The sample was then dried using hot air oven at 50 °C for 12 hours to obtain purified crude polysaccharide powder.

#### Purification of crude polysaccharides using liquid-liquid extraction

The purification of Chaga mushroom polysaccharides was performed according to Hu et al., 2016^35^ with slight modifications. The crude polysaccharide (5 g) previously obtained after adding Sevag reagent was dissolved in distilled water (250 mL w/v), filtered through a 0.45-µm Millipore filter, and subsequently, the solution was loaded onto an anion-exchange DEAE-cellulose column (2.6 cm × 50 cm) equilibrated with distilled water. The column was eluted with distilled water and 0.3 M NaCl, respectively. The eluent was collected at a flow rate of 1.00 mL/min. Four mL fractions were collected in a single tube repeatedly, which were further fractionated by size-exclusion chromatography on a Sephadex G-100 column (2.6 cm × 70 cm) and eluted with deionized water. Each fraction was monitored using the phenol-sulphuric acid method for the determination of the carbohydrate content at 490 nm with glucose standard and subsequently lyophilized in a lyophilizer at −60 °C for overnight.

#### GC-MSMS analysis for monosaccharide composition analysis

As polysaccharides are non-polar and non-volatile in nature, first they were hydrolyzed using trifluoroacetic acid (TFA) to derivitize into monosaccharides^35^. Briefly, 30 mg of purified lyophilized polysaccharides were hydrolyzed to monosaccharides in 3 mL of 2 M trifluoro acetic acid (TFA) in a 5 mL tube, and kept at 120 °C for 20 mins, followed by evaporation under reduced pressure (40 °C). The obtained powder was treated with trimethylsilylation reagent, TMS (N-Trimethylsilylimidazole). Briefly,10 mg of the sample was weighed and excess silylating reagent (1:4) N-Trimethylsilylimidazole and pyridine (2:1 molar ratio of reagent to active hydrogen) was added. The solution was incubated at 25 °C for 15 mins, and filtered through 0.45 μm filter, and finally injected into GC-MSMS (7890B GC-240 Ion trap MS, Agilent technologies, USA) using pyridine solvent. Xylose, glucose, arabinose, rhamnose, mannose, and galactose were used as internal standards for GC-MSMS analysis. The capillary column VF-5MS (Length-30m, ID-0.25 mm, Film-0.25 µm, maximum temp-325 °C) was used for phase separation. Helium was used as the carrier gas at a flow rate of 1.0 mL/min. The temperature was programmed as follows: initial temperature 110 °C, maintained for a min; rose up to 300 °C at a rate of 15 °C/min, and maintained for 20 mins. The total analysis time was 25 min and the equilibration time was 2 min. The temperature of the injection port was 250 °C and 1 μL sample was injected in splitless mode. The mass spectrometer was operated in electron ionization mode at an ionizing energy of 70 eV, the temperature of ion source 250 °C, and scanning was performed from m/z 50 to 500. The molar ratio of the monosaccharides present in the polysaccharide extract was calculated using the area normalization method.

### Zebrafish experiments

#### Zebrafish rearing and polysaccharide exposure

All the methods were approved and carried out in accordance with relevant guidelines and regulations of the Institutional Ethical Committee (IEC) of KIIT University. Adult wild-type zebrafish were maintained in a standard flow through system (28.0 ± 1 °C, 12 h:12 h dark/light cycle), and fed a protein-rich diet twice daily. Zebrafish embryos were obtained from mating conducted in the laboratory using 2:1 female: male ratio, and the embryos were collected after spawn and washed thrice with embryo medium. The culture density per mating was around 80 healthy embryos. The fertilized and normal embryos at 3-hour post-fertilization (hpf) were randomly distributed and staged in 6-well microplates for the exposure experiment after inspection under an inverted microscope (EVOS, Thermo Fishcer SCIENTIFIC). Zebrafish embryos of 6 hpf (n = 320), in the shield stage and initiating organogenesis, were exposed to different concentrations of the purified lyophilized polysaccharides (5 mg/mL, 2.5 mg/mL, and 1 mg/mL) and embryo water (0.06% sea salt with methylene blue), which was taken as the control (n = 20/each group). The exposure time was 24 hours, followed by replacement with fresh embryo water daily for the rest of the development period. All the experiments were performed in triplicates. The petridish containing treated and untreated embryos were kept at 28 ± 1.0 °C in 14/10 hours light and dark condition for 7 days. Early developmental stages were recorded up to 5-day post-fertilization (dpf) by anesthetizing the fish in 300 mg/L tricaine methanesulfonate (MS-222). Morphological deformities during embryonic development were recorded, which included several phenotypic observations; body length, otic vesicle, heart, swim bladder, yolk sac, and craniofacial morphology. Hatching rate, viability rate, and heart beats rate were also measured using a stereomicroscope (Nikon, Japan).

#### Cell cycle analysis using flow cytometry

Cell cycle analysis was performed in the zebrafish larvae (3 dpf) exposed to Chaga mushroom polysaccharides (2.5 mg/mL) using propidium iodide staining followed by flow cytometry. This analysis will reveal whether Chaga mushroom polysaccharides has any effect on the different stages of cell cycle (G1, S, G2). Cell cycle analysis is based on the measurement of DNA content of the cell by fluorescent labelling of the cell, followed by excitation of the laser beam that will pass through it and emitted light of different wavelength that can be measured and quantified. For this, fresh zebrafish larvae (3 dpf, n = 20 each for control and test groups) were exposed to Chaga mushroom polysaccharides for 24 hours, thoroughly sonicated, and homogenized in phosphate buffer saline (PBS). The homogenate was then mixed with a staining solution containing Triton X-100, RNase A, and propidium iodide (PI), followed by filtration using 70-micron strainer and flow cytometry analysis at an excitation wavelength of around 480 nm (blue light) and emission wavelength at 620 nm (red light) in a BD FACS Canto II flow cytometer (BD Biosciences, USA). All the experiments were performed in triplicates. The data obtained was analyzed using FACS software and the percentage of cells in the G1, S, and G2/M phases of cell cycle was estimated accordingly. Specifically, at first, we used FSC versus SSC gating to center the cells. Then, we used FSC height (y) versus FSC area (x) to discriminate the doublets from the singlets so that only single cells were selected for the generating the histogram (Supplementary Figs. 1 and 2). This strategy efficiently excludes the doublets (2n + 2n cells), and thus precludes the false peaks (mainly the G2) that can be generated due to the selection of doublet cells.

#### Acridine orange staining

Qualitative apoptosis analysis in the zebrafish embryos exposed to the Chaga mushroom polysaccharides was performed using acridine orange (AO) staining. AO, a nucleic-specific metachromatic dye, interacts with DNA and RNA by intercalating or electrostatic attraction and deeply stains necrotic or very late apoptotic cells. AO when bound to double strand DNA emits green fluorescence and when bound to single strand DNA or single strand RNA emits red fluorescence. This unique characteristic makes acridine orange useful for cell apoptosis studies. Briefly, untreated and polysaccharide-exposed (for 24 hours at 6 hpf) zebrafish larvae of 5 dpf (n = 20/group) were stained with 5 μg/mL acridine orange for 20 min. Then, the excess stain was removed by washing with embryo water. All the experiments were performed in triplicates. Images were taken in the green channel of EVOS inverted fluorescent microscope (Thermo Fischer Scientific, USA) to assess the apoptosis by comparing the number of apoptotic cells (green dots) in different zebrafish embryos.

### Apoptosis analysis using flow cytometry

Quantitative analysis of apoptosis in the single cells of zebrafish embryos was done using FITC Annexin V/Dead Cell Apoptosis kit (Invitrogen). Using recombinant Annexin V conjugated to green-fluorescent FITC dye, the kit detects the internalization of phosphatidyl-serine in the apoptotic cells, and dead cells are detected using propidium iodide (PI) that binds to exposed DNA. Briefly, untreated and polysaccharide-treated (for 24 hours at 6 hpf) zebrafish larvae of 5 dpf (n = 10/group) were homogenized thoroughly by sonication independently for single cell isolation. Then the cell supernatant was filtered through 70-micron strainer and stained using FITC-Annexin V/PI and analyzed in a BD FACS Canto II flow cytometer. All the experiments were performed in triplicates.

### Intra-cellular ROS analysis

For intra-cellular (reactive oxygen species) ROS quantification, the negatively charged oxidized form of ROS indicator, carboxy-H_2_DCFDA was utilized followed by flow cytometry analysis. The cell-permeant H_2_DCFDA passively diffuses into the cells and is retained at the intracellular level after cleavage by intracellular esterase. Upon oxidation by ROS, the non-fluorescent H_2_DCFDA is converted to the highly fluorescent 2’,7’-dichlorofluorescein (DCF). Briefly, 20 zebrafish larvae of 5 dpf exposed to different concentrations of Chaga mushroom polysaccharide (1 mg/mL, 2.5 mg/mL and 5 mg/mL) for 24 hours at 6 hpf were independently homogenized by sonication for single cell isolation, filtered through 70-micron strainer and stained with H_2_DCFDA, followed by flow cytometric analysis using BD FACS Canto II flow cytometer. All the experiments were performed in triplicates.

### Metabolic enzyme assays

In order to study the effect of stress on the metabolic pathways, glutathione-S-transferase assay (GST) was performed. Briefly, about 5 zebrafish were selected from each group (5 dpf), homogenized in embryo water and were assayed for the analysis of the respective enzymes using the manufacturer’s instructions. GST activity was expressed as µmole/mL/min. All spectrophotometric measurements were performed using the Shimadzu UV-1800 UV spectrophotometer at wavelengths ranging from 340-400 nm. The experiments were performed in triplicates for confirmatory results.

### Ethical statement

All the methods were approved and carried out in accordance with relevant guidelines and regulations of the Institutional Ethical Committee (IEC) of KIIT University.

## Supplementary information

Supplementary information.

Supplementary figure 1.

Supplementary figure 2.
