# Supplementary material for: Molecular insights and cell cycle assessment upon exposure to Chaga (Inonotus obliquus) mushroom polysaccharides in zebrafish (Danio rerio)
Source: Sci Rep. 2020 May 4;10:7406. doi: 10.1038/s41598-020-64157-3 (PMC7198532; doi:10.1038/s41598-020-64157-3)
Supplement: Supplementary file 1 — Supplementary information. [file 41598_2020_64157_MOESM1_ESM.docx]

Molecular insights and cell cycle assessment upon exposure to Chaga (*Inonotus obliquus*) mushroom polysaccharides in zebrafish

Authors:

Jehane Ibrahim Eid*^1^, Biswadeep Das^2^

^1^Department of Zoology, Cairo University, Egypt 12613.

^2^School of Biotechnology, KIIT University, Bhubaneswar 751024, India

Correspondence:

*Department of Zoology, Cairo University, Egypt 12613

Ph: +201011439568

jehaneeid@sci.cu.edu.eg

Supplementary Figure 1:

Cell cycle assessment using flow cytometry showing the number of zebrafish embryo cells (3 dpf) in the different phases of the cell cycle (G1-S-G2) without any polysaccharide exposure.

Supplementary Figure 2:

Cell cycle assessment using flow cytometry showing the number of zebrafish embryo cells (3 dpf) grown in chaga mushroom polysaccharides (2.5 mg/mL).
