# Supplementary figures and images for: Molecular insights and cell cycle assessment upon exposure to Chaga (Inonotus obliquus) mushroom polysaccharides in zebrafish (Danio rerio)
Source: Sci Rep. 2020 May 4;10:7406. doi: 10.1038/s41598-020-64157-3 (PMC7198532; doi:10.1038/s41598-020-64157-3)

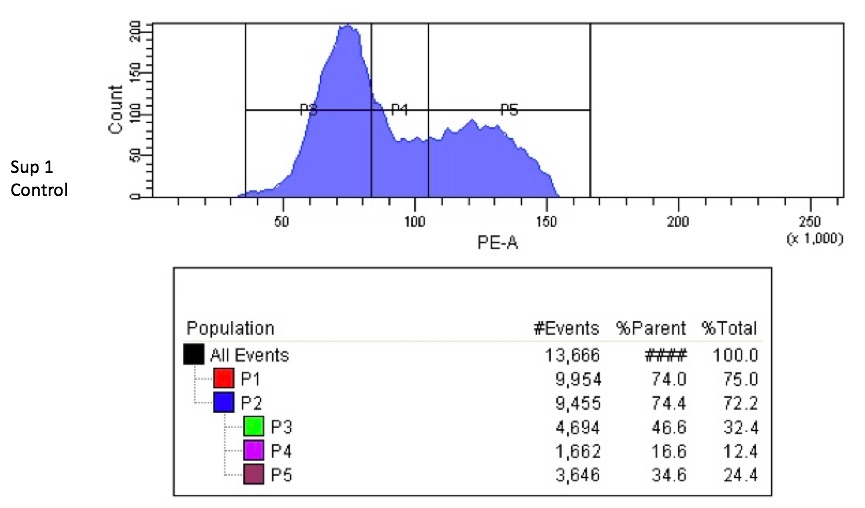

Supplement: Supplementary file 2 — Supplementary figure 1. [file 41598_2020_64157_MOESM2_ESM.jpg]

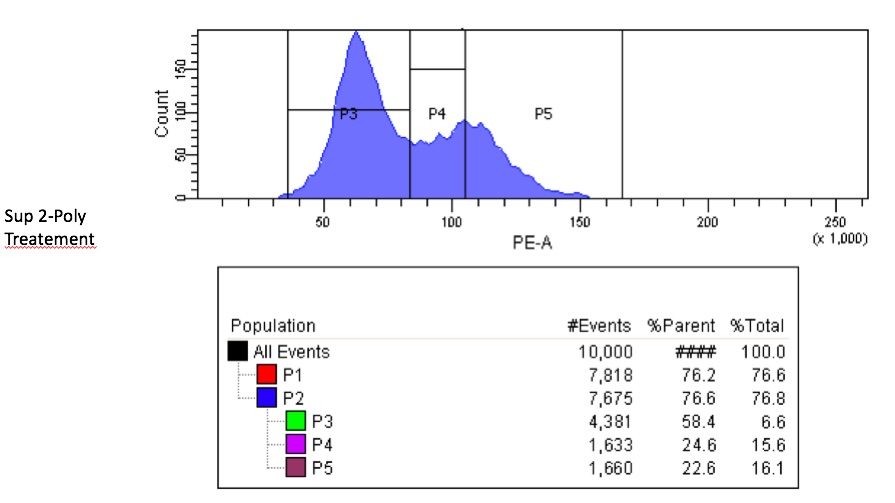

Supplement: Supplementary file 3 — Supplementary figure 2. [file 41598_2020_64157_MOESM3_ESM.jpg]
